# Longitudinal Trajectories of the Atherogenic Index of Plasma and Their Associations with Clinical Outcomes After Transcatheter Aortic Valve Replacement

**DOI:** 10.3390/jcm15197448

**Published:** 2026-09-24

**Authors:** Lihan Wang, Wenjing Sheng, Chengyi Zhang, Qifeng Zhu, Boren Tan, Feiyu Wu, Xianbao Liu

**Affiliations:** 1 Department of Cardiology, The Second Affiliated Hospital, School of Medicine, Zhejiang University, Hangzhou 310009, China; 12618445@zju.edu.cn (L.W.); 22318381@zju.edu.cn (W.S.); 12618441@zju.edu.cn (C.Z.); zhuqifeng@zju.edu.cn (Q.Z.); 22518361@zju.edu.cn (B.T.); 22418421@zju.edu.cn (F.W.); 2 State Key Laboratory of Transvascular Implantation Devices, Hangzhou 310009, China; 3 Heart Regeneration and Repair Key Laboratory of Zhejiang Province, Hangzhou 310009, China; 4 Transvascular Implantation Devices Research Institute, Hangzhou 310053, China; 5 Binjiang Institute of Zhejiang University, Hangzhou 310053, China

**Keywords:** transcatheter aortic valve replacement, atherogenic index of plasma, group-based trajectory modeling, clinical outcomes, valve hemodynamics

## Abstract

**Background/Objectives:** The atherogenic index of plasma (AIP) reflects atherogenic dyslipidemia, but the clinical relevance of its longitudinal patterns after transcatheter aortic valve replacement (TAVR) remains unclear. We aimed to characterize AIP trajectories and explore their associations with clinical outcomes and echocardiographic changes after TAVR. **Methods:** This retrospective analysis included 1548 patients with at least two AIP measurements from within 7 days before TAVR through follow-up. Group-based trajectory modeling was used to identify longitudinal AIP patterns. Cox proportional hazards models and linear mixed-effects models were applied to assess clinical outcomes and longitudinal echocardiographic changes, respectively. Because trajectory assignment incorporated post-TAVR measurements, the analyses were considered exploratory. **Results:** Three distinct AIP trajectories were identified: low-level (*n* = 431, 27.8%), intermediate-level (*n* = 807, 52.1%), and high-level (*n* = 310, 20.0%). Higher trajectories were characterized by less favorable cardiometabolic profiles. Compared with the low-level trajectory, neither the intermediate-level trajectory (adjusted HR 0.83, 95% CI 0.60–1.16; *p* = 0.277) nor the high-level trajectory (adjusted HR 1.09, 95% CI 0.74–1.61; *p* = 0.663) was significantly associated with major adverse cardiovascular and cerebrovascular events. No significant associations with all-cause mortality or longitudinal echocardiographic recovery were detected. **Conclusions:** Distinct longitudinal AIP trajectories characterized different cardiometabolic profiles after TAVR, but no statistically significant association with major clinical outcomes or echocardiographic recovery was detected. These findings should be interpreted cautiously given temporal overlap in trajectory ascertainment, selection related to repeated measurements, and limited event numbers.

## 1. Introduction

Transcatheter aortic valve replacement (TAVR) has become a standard therapeutic strategy for patients with severe aortic valve disease (AVD) across a broad spectrum of surgical risk profiles [1,2,3]. With expanding indications and improving procedural outcomes, increasing attention has shifted toward long-term outcomes after TAVR, including residual cardiovascular risk, cardiac remodeling, and prosthetic valve performance [4,5,6]. Although TAVR effectively relieves left ventricular pressure overload and improves symptoms, a proportion of patients continue to experience adverse clinical events during follow-up, and variability exists in postprocedural cardiac recovery [5,7]. The factors associated with this heterogeneity in long-term clinical and echocardiographic outcomes after TAVR remain incompletely characterized.

The pathophysiology of AVD shares several biological characteristics with atherosclerosis, including lipid deposition, chronic inflammation, oxidative stress, and progressive calcification [8,9,10,11,12]. These overlapping mechanisms suggest that lipid-related metabolic abnormalities may be involved in the development and progression of valvular disease and may also reflect systemic cardiovascular vulnerability in patients undergoing TAVR [13,14,15,16]. The atherogenic index of plasma (AIP), calculated as the logarithmic ratio of triglycerides to high-density lipoprotein cholesterol (HDL-C), is a composite marker reflecting atherogenic dyslipidemia [17,18]. Elevated AIP has been associated with increased cardiovascular risk and adverse outcomes in several clinical populations [19,20,21]. However, the clinical relevance of AIP in patients undergoing TAVR remains uncertain, particularly because TAVR substantially alters the hemodynamic environment by relieving valvular obstruction.

Most previous studies evaluating lipid-related markers in TAVR populations have relied on measurements obtained at a single time point, usually before the procedure [16,19]. Such an approach may not fully capture the dynamic nature of lipid metabolism during the peri-procedural and follow-up periods. Metabolic profiles are dynamic and may change over time as a result of aging, comorbidities, medical therapy, nutritional status, and postprocedural clinical course. Therefore, longitudinal assessment of lipid-related biomarkers may provide complementary information beyond conventional baseline measurements. Longitudinal trajectory modeling provides an alternative approach by characterizing patterns of biomarker change over time rather than relying exclusively on a single baseline measurement [20,22,23,24,25]. However, longitudinal patterns of AIP in patients undergoing TAVR and their associations with clinical outcomes and cardiac remodeling remain poorly understood. Therefore, the present study aimed to identify distinct longitudinal AIP trajectories in patients undergoing TAVR, characterize their clinical and metabolic profiles, and explore their associations with clinical outcomes and longitudinal echocardiographic changes.

## 2. Methods

### 2.1. Study Population

This study was a retrospective secondary analysis of deidentified data from the Transcatheter Aortic Valve Replacement Single-Center Registry in the Chinese Population (TORCH registry; ClinicalTrials.gov identifier: NCT02803294). The TORCH registry is a single-center institutional registry established at the Second Affiliated Hospital, School of Medicine, Zhejiang University, to systematically collect clinical, procedural, laboratory, imaging, and follow-up data from patients undergoing TAVR. The registry includes consecutive patients treated with TAVR and maintains structured longitudinal follow-up through outpatient visits and telephone interviews.

Between March 2013 and September 2025, a total of 2068 patients with severe AVD who underwent TAVR were screened for eligibility. The inclusion criteria were as follows: (1) severe AVD, including severe aortic stenosis (AS) and/or moderate-to-severe or severe aortic regurgitation (AR), confirmed by echocardiography; (2) eligibility for TAVR based on anatomical and technical assessment; and (3) provision of written informed consent and agreement for follow-up.

Patients were excluded if they met any of the following criteria: (1) missing follow-up information; (2) unavailable baseline AIP measurements; (3) fewer than two available AIP measurements obtained from 7 days before the index procedure through follow-up; or (4) occurrence of catastrophic periprocedural complications, including coronary rupture, aortic dissection, or conversion to open surgery.

A total of 1548 patients were included in the final trajectory analysis (Figure 1). To assess potential selection related to trajectory ascertainment, baseline characteristics were compared between patients included in and excluded from the trajectory analysis. In addition, among patients with available baseline AIP measurements, baseline characteristics were compared between those included in the trajectory analysis and those without sufficient longitudinal AIP measurements for trajectory classification. The TORCH registry was conducted in accordance with the Declaration of Helsinki and was approved by the Human Research Ethics Committee of the Second Affiliated Hospital, Zhejiang University School of Medicine (approval number: 2016 Ethical Review No. 032; project acceptance number: Yan 2016-037; event acceptance number: I2016001063; approval date: 6 July 2016). The requirement for additional follow-up ethical review for the present retrospective analysis of TORCH registry data covering the 2016–2025 period was waived by the Human Research Ethics Committee of the Second Affiliated Hospital, Zhejiang University School of Medicine. The registry was registered at ClinicalTrials.gov (identifier: NCT02803294). Written informed consent for registry participation, use of clinical data, and follow-up was obtained from all participants. The present analysis used deidentified registry data within the scope of the approved registry protocol.

### 2.2. Data Collection and Definitions

AIP was calculated as log_10_(triglycerides/HDL-C), with lipid concentrations expressed in molar units [17]. Serial AIP measurements were obtained within 7 days before TAVR and during subsequent follow-up visits according to routine clinical practice. Detailed baseline and longitudinal medication data, including the type and dose of lipid-lowering agents, treatment initiation or discontinuation, subsequent treatment modifications, and medication adherence, were not systematically captured throughout the entire registry period and were therefore not included in the present analysis.

Transthoracic echocardiography was performed at baseline, at discharge or 30 days after TAVR, and during follow-up according to institutional protocols. Echocardiographic parameters included peak aortic jet velocity (Vmax), mean transvalvular gradient (PGmean), aortic valve area, left ventricular ejection fraction (LVEF), and left ventricular mass index (LVMI). Valvular regurgitation, including AR, mitral regurgitation (MR), tricuspid regurgitation (TR), and prosthetic valve regurgitation, was also assessed.

Prosthetic valve function and valve-related outcomes were assessed according to the Valve Academic Research Consortium-3 (VARC-3) criteria. Hemodynamic valve deterioration was defined based on changes in transvalvular gradients, effective orifice area, and intraprosthetic regurgitation occurring ≥90 days after TAVR. Bioprosthetic valve failure (BVF) was defined as irreversible severe valve dysfunction (stage 3), aortic valve reintervention, or valve-related death [26].

### 2.3. Outcome Definitions

Clinical outcomes included major adverse cardiovascular and cerebrovascular events (MACCEs) and all-cause mortality. MACCEs were defined as a composite of cardiovascular death, myocardial infarction, stroke, hospitalization for heart failure, or BVF. Follow-up assessments were conducted at 30 days and annually thereafter by a dedicated follow-up team through outpatient visits or structured telephone interviews. Follow-up duration was calculated from the date of the index TAVR procedure to the occurrence of an outcome event or the last available follow-up.

### 2.4. Statistical Analysis

The analytical framework was designed to (1) identify longitudinal AIP trajectory patterns, (2) characterize their clinical and metabolic profiles, and (3) explore their associations with clinical outcomes and postprocedural hemodynamic changes.

Because AIP trajectory groups were derived from longitudinal measurements extending into the follow-up period, the present analyses were considered primarily exploratory and descriptive. Accordingly, trajectory groups were interpreted as post hoc classifications reflecting longitudinal metabolic patterns rather than baseline-defined exposures.

Continuous variables are presented as mean ± standard deviation (SD) for normally distributed data or median (interquartile range [IQR]) for non-normally distributed data. Categorical variables are expressed as counts and percentages. Comparisons across the three AIP trajectory groups were performed using one-way analysis of variance or the Kruskal–Wallis test for continuous variables, as appropriate, and the chi-square test or Fisher’s exact test for categorical variables. For comparisons between two groups in the supplementary selection analyses, Student’s *t*-test or the Mann–Whitney U test was used for continuous variables, as appropriate.

#### 2.4.1. AIP Trajectory Analysis

Group-based trajectory modeling (GBTM) was applied to identify distinct longitudinal patterns of AIP over time. Longitudinal AIP data were initially processed in R and subsequently analyzed in Stata using the *traj* plugin (Bobby L. Jones; available at: https://www.andrew.cmu.edu/user/bjones/traj/; accessed on 31 January 2026). Models specifying two to five trajectory groups were fitted and compared. Model selection was based on the Bayesian Information Criterion (BIC), Akaike Information Criterion (AIC), log-likelihood, entropy, minimum group size, and clinical interpretability. Entropy >0.7 was considered to indicate acceptable classification quality, and a minimum trajectory group size of >5% was prespecified to avoid unstable small-class solutions. Each trajectory was modeled using polynomial functions of time, with the order of the polynomial terms determined iteratively to achieve the best model fit. Patients were assigned to trajectory groups according to the maximum posterior probability. The three-group model was selected as a parsimonious solution with adequate discrimination and clinically interpretable trajectory patterns.

#### 2.4.2. Clinical Outcomes Analysis

Cumulative event rates across AIP trajectory groups were estimated using the Kaplan–Meier method and compared using the log-rank test. Cox proportional hazards models were constructed to explore the associations between baseline AIP and AIP trajectory groups and clinical outcomes, with results reported as hazard ratios (HRs) and 95% confidence intervals (CIs). In the primary trajectory-analysis cohort (*n* = 1548), baseline AIP was analyzed as a continuous variable, whereas AIP trajectory group was analyzed as a categorical exposure, with Traj1 as the reference group. The proportional hazards assumption was assessed using Schoenfeld residuals.

Three Cox regression models were constructed. The crude model was unadjusted. Model I was adjusted for age and sex. Model II was additionally adjusted for New York Heart Association (NYHA) class III/IV, hypertension, and diabetes mellitus. To preserve temporal ordering, postprocedural variables were not included in the adjusted Cox models. As a complementary analysis addressing potential selection related to longitudinal trajectory ascertainment, baseline AIP was additionally evaluated in the broader cohort of 1628 patients with available baseline AIP measurements, irrespective of eligibility for trajectory classification. Baseline AIP was analyzed both continuously and by tertiles using the same Cox regression adjustment strategy as the primary analysis. Subgroup analyses were performed across prespecified variables, and interaction terms were tested within the Cox proportional hazards models. Given the number of subgroup comparisons, these analyses were considered exploratory.

#### 2.4.3. Longitudinal Echocardiographic Analysis

Longitudinal changes in echocardiographic parameters, including Vmax, PGmean, LVEF, and LVMI, were analyzed using linear mixed-effects models (LMMs). Trajectory group, follow-up visit treated as a categorical variable, and their interaction were included as fixed effects, with a patient-level random intercept to account for within-subject correlation. An unstructured covariance matrix was specified, and models were adjusted for baseline values of each corresponding echocardiographic parameter. Type III tests were used to assess the overall effects of trajectory group, time, and their interaction. Model-derived estimated marginal means with 95% CIs were used to illustrate longitudinal trends across trajectory groups. All available echocardiographic observations were included, allowing unequal numbers of measurements across patients and follow-up visits. No single-value imputation was performed for missing echocardiographic data. Likelihood-based estimation assumes that missing observations are missing at random conditional on the observed data, although informative missingness cannot be excluded.

All statistical analyses were performed using Stata version 18.0 (StataCorp, College Station, TX, USA) and R version 4.5.2 (R Foundation for Statistical Computing, Vienna, Austria). All tests were two-sided, and a *p* value <0.05 was considered statistically significant.

## 3. Results

### 3.1. Study Population and AIP Trajectory Classification

A total of 1548 patients undergoing TAVR were included in the final analysis (Figure 1). GBTM identified three distinct longitudinal AIP trajectory groups (Figure 2; Supplementary Table S1). Among candidate models, the three-group solution was selected based on model fit, discrimination, and clinical interpretability, with an entropy of 0.780 and adequate separation between the groups. The proportions of patients assigned to Traj1, Traj2, and Traj3 were 27.8% (431/1548), 52.1% (807/1548), and 20.0% (310/1548), respectively. These trajectories corresponded to persistently low AIP, stable intermediate AIP, and relatively high AIP with a gradual decreasing trend over time.

Compared with patients included in the trajectory analysis, excluded patients had a generally higher-risk clinical profile, including older age, higher STS scores, and a greater prevalence of several comorbidities, although baseline AIP was similar between groups (*p* = 0.981; Supplementary Table S2). Among patients with available baseline AIP, most baseline characteristics and lipid parameters were similar between those included in and excluded from trajectory classification, although several differences remained (Supplementary Table S3).

### 3.2. Baseline Characteristics According to AIP Trajectory Groups

Baseline characteristics differed across the AIP trajectory groups (Table 1). Patients in Traj3 were younger than those in Traj1 and Traj2 (75 [IQR, 70–80] vs. 75 [IQR, 70–79] vs. 73 [IQR, 68–78] years, respectively; *p* < 0.001) and had higher body mass index (BMI) and body surface area (BSA) (both *p* < 0.001). The proportion of men decreased across increasing AIP trajectory groups (63.1% vs. 59.0% vs. 53.9%; *p* = 0.041), and STS scores were modestly lower in Traj3 (3.32 [IQR, 2.06–5.90] vs. 3.10 [IQR, 1.83–5.34] vs. 2.90 [IQR, 1.75–4.79] %; *p* = 0.015). Several cardiometabolic comorbidities were more prevalent in Traj3, including dyslipidemia (11.4% vs. 15.9% vs. 43.2%; *p* < 0.001), diabetes mellitus (13.9% vs. 16.1% vs. 27.1%; *p* < 0.001), hypertension (54.1% vs. 57.9% vs. 63.5%; *p* = 0.036), and coronary artery disease (7.2% vs. 9.7% vs. 12.9%; *p* = 0.034). In contrast, chronic kidney disease was less frequent in Traj3 (57.1% vs. 54.0% vs. 41.3%; *p* < 0.001). Baseline lipid profiles were consistent with trajectory classification, with progressively higher triglycerides (0.74 vs. 1.07 vs. 1.71 mmol/L) and low-density lipoprotein cholesterol (LDL-C) levels (1.81 vs. 2.01 vs. 2.23 mmol/L) and lower HDL-C levels (1.43 vs. 1.17 vs. 0.98 mmol/L) from Traj1 to Traj3 (all *p* < 0.001). The median baseline AIP values were −0.29, −0.03, and 0.23 in Traj1, Traj2, and Traj3, respectively (*p* < 0.001).

Baseline echocardiographic parameters were generally comparable across trajectories, although modest differences were observed in PGmean (45 [IQR, 30–64] vs. 43 [IQR, 24–57] vs. 44 [IQR, 34–59] mmHg; *p* = 0.028), Vmax (4.39 [IQR, 3.63–5.16] vs. 4.31 [IQR, 3.29–4.89] vs. 4.38 [IQR, 3.98–4.97] m/s; *p* = 0.018), LVEF (60.5 [IQR, 53.4–65.9] vs. 59.8 [IQR, 48.1–65.3] vs. 61.0 [IQR, 52.8–66.2] %; *p* = 0.014), and moderate or greater TR (17.6% vs. 9.8% vs. 6.8%; *p* < 0.001). Computed tomography-derived anatomical characteristics were largely comparable across groups, except for aortic angulation (*p* < 0.001).

### 3.3. Procedural Characteristics and Clinical Outcomes

Procedural characteristics were generally comparable across the AIP trajectory groups (Table 2). Transfemoral access was used in 89.1% of patients, with no significant difference across groups (*p* = 0.408). The distribution of implanted valve types, annular oversizing ratios, and the use of pre- or post-dilatation were similar among the three groups (all *p* > 0.05).

At discharge, disabling stroke occurred more frequently in Traj3 than in the other groups (0.7% vs. 0.2% vs. 1.6% in Traj1, Traj2, and Traj3, respectively; *p* = 0.026), whereas the overall stroke rate and other complications, including major vascular complications, severe bleeding, new AF, and permanent pacemaker implantation, showed no significant differences. At 30 days, all-cause mortality remained low (0.1% overall). Stroke occurred in 4.8%, 2.0%, and 2.8% of patients in Traj3, Traj2, and Traj1, respectively (*p* = 0.034), and disabling stroke occurred in 1.9%, 0.2%, and 0.7%, respectively (*p* = 0.011).

During follow-up, overall event rates remained low across all trajectory groups (Table 2). At 1 year, all-cause mortality was 0.9% in Traj1, 0.7% in Traj2, and 2.3% in Traj3 (*p* = 0.109), with no significant difference in cardiovascular mortality (*p* = 0.825). Non-cardiovascular mortality differed across groups (0.2% vs. 0.2% vs. 1.6%, respectively; *p* = 0.019). The overall stroke rate at 1 year did not differ significantly among trajectory groups (3.5% vs. 3.0% vs. 5.8%; *p* = 0.076), although disabling stroke occurred in 0.9%, 0.4%, and 2.3% of patients in Traj1, Traj2, and Traj3, respectively (*p* = 0.013). Other 1-year clinical outcomes, including myocardial infarction, heart failure rehospitalization, and BVF, were comparable across trajectory groups (all *p* > 0.05; Table 2).

Kaplan–Meier analyses demonstrated no significant differences in cumulative incidence of MACCEs (*p* = 0.499) or all-cause mortality (*p* = 0.692) across the AIP trajectory groups throughout follow-up (Figure 3).

### 3.4. Association Between AIP Trajectories and Clinical Outcomes

In multivariable Cox models, no statistically significant association between AIP trajectory group and MACCEs was detected (Table 3). Compared with Traj1, neither Traj2 (Model II: HR, 0.83; 95% CI, 0.60–1.16; *p* = 0.277) nor Traj3 (HR, 1.09; 95% CI, 0.74–1.61; *p* = 0.663) was significantly associated with MACCEs. Within the trajectory-analysis cohort, baseline AIP analyzed as a continuous variable was not significantly associated with MACCEs (HR, 0.84; 95% CI, 0.47–1.49; *p* = 0.557).

AIP trajectory groups were not significantly associated with all-cause mortality, cardiovascular mortality, stroke, heart failure rehospitalization, or BVF across the adjusted models. Within the trajectory-analysis cohort, baseline AIP was inversely associated with cardiovascular mortality (Model II: HR, 0.10; 95% CI, 0.01–0.62; *p* = 0.014), whereas its association with all-cause mortality did not reach statistical significance after adjustment (HR, 0.34; 95% CI, 0.11–1.02; *p* = 0.055; Table 3).

In the complementary analysis conducted in the broader cohort of 1628 patients with available baseline AIP measurements, irrespective of eligibility for trajectory classification, baseline AIP was not significantly associated with MACCEs when analyzed continuously (Model II: HR, 0.75; 95% CI, 0.43–1.30; *p* = 0.309) or by tertiles (Supplementary Table S4). Baseline AIP was also not significantly associated with stroke, heart failure rehospitalization, or BVF. In contrast, higher baseline AIP was inversely associated with all-cause and cardiovascular mortality.

### 3.5. Longitudinal Echocardiographic Changes

At discharge and 30 days, aortic valve hemodynamic parameters were generally comparable across the AIP trajectory groups. LVEF differed among groups at discharge (*p* = 0.012), whereas moderate or greater PPM at 30 days was comparable across groups (*p* = 0.333). Moderate or greater TR differed at both discharge and 30 days (*p* = 0.001 for both), with the highest prevalence observed in Traj1. During long-term follow-up, most echocardiographic parameters remained comparable across groups. Isolated between-group differences were observed in LVMI at 2 years (*p* = 0.034), LVEF and moderate or greater AR at 3 years (*p* = 0.023 and *p* = 0.030, respectively), moderate or greater TR at 4 years (*p* < 0.001), and Vmax and PGmean at 5 years (*p* = 0.022 and *p* = 0.030, respectively) (Supplementary Table S5).

Across all trajectory groups, serial echocardiographic assessments showed improvements in valve hemodynamics and left ventricular remodeling after TAVR (Figure 4 and Supplementary Table S5). Vmax decreased from 4.35 m/s at baseline to approximately 2.00–2.20 m/s during follow-up, while PGmean declined from 44 mmHg to approximately 8–12 mmHg. LVEF increased and LVMI decreased over time across the trajectory groups.

Linear mixed-effects models demonstrated no significant differences among trajectory groups in longitudinal changes in peak aortic velocity (*p* = 0.578), PGmean (*p* = 0.177), or LVMI (*p* = 0.475) over time. A difference in LVEF trajectories did not reach statistical significance (*p* = 0.062) (Figure 4).

### 3.6. Subgroup Analysis

Subgroup analyses showed no significant interaction between AIP trajectories and most baseline or procedural characteristics (Figure 5). A statistically significant interaction was observed according to the presence of moderate or greater AR (*P* for interaction = 0.027). Among patients with moderate or greater AR, Traj3 was associated with a higher hazard of MACCEs compared with Traj1 (HR 2.00; 95% CI: 1.15–3.46; *p* = 0.014). No significant associations were observed for Traj2 versus Traj1 or Traj3 versus Traj1 in the other prespecified subgroups.

## 4. Discussion

### 4.1. Principal Findings

In this study, we characterized longitudinal AIP trajectories in a large cohort of patients undergoing TAVR and examined their associations with clinical outcomes and longitudinal echocardiographic changes. Several key findings emerged. First, three distinct AIP trajectories were identified, comprising low-level, intermediate-level, and high-level patterns, with progressively less favorable lipid and cardiometabolic profiles across the groups. Second, despite these differences in metabolic characteristics, no statistically significant associations between AIP trajectory groups and MACCEs, all-cause mortality, or other individual clinical outcomes were detected in multivariable analyses. Third, TAVR was associated with substantial improvements in valve hemodynamics and left ventricular remodeling across all trajectory groups, without significant trajectory-related differences in longitudinal echocardiographic changes. In the complementary baseline AIP analysis, baseline AIP was not associated with MACCEs, although inverse associations with mortality were observed. Collectively, these findings suggest that longitudinal AIP patterns can distinguish different metabolic phenotypes but did not demonstrate clear associations with post-TAVR clinical outcomes or echocardiographic recovery in this cohort.

### 4.2. AIP Trajectories and Metabolic Phenotyping

AIP integrates triglyceride and HDL-C concentrations into a composite measure of atherogenic dyslipidemia and has been associated with cardiovascular risk in several clinical populations [17,18,19,20,21]. In the present study, trajectory modeling extends this concept by incorporating temporal dynamics, thereby distinguishing persistent metabolic phenotypes rather than relying on single-point measurements [22,23,24,25].

Patients in higher AIP trajectory groups exhibited a consistent clustering of metabolic abnormalities, including higher triglyceride levels, lower HDL-C levels, and a greater prevalence of diabetes mellitus and dyslipidemia [22,23,24,25]. This internal consistency supports the biological validity of trajectory classification. Importantly, this trajectory-based framework extends beyond static risk stratification and provides a more integrative representation of longitudinal metabolic patterns [19,20,24]. However, the metabolic differences across trajectory groups were not accompanied by statistically significant differences in long-term clinical outcomes after adjustment for prespecified baseline clinical covariates. This discordance suggests that the clinical relevance of atherogenic dyslipidemia may be context-dependent and potentially attenuated in the setting of advanced valvular disease treated with valve intervention [8,14].

### 4.3. Lipid-Related Mechanisms Across Disease Stages

Accumulating evidence supports a role for lipid infiltration, oxidative stress, inflammatory signaling, and osteogenic differentiation of valvular interstitial cells in the initiation and progression of calcific AVD [11,12,13,14,15]. These mechanisms are thought to contribute to early valve remodeling and calcification. Within this conceptual framework, AIP may serve as an integrative marker of atherogenic dyslipidemia and lipid-related cardiovascular vulnerability.

However, randomized trials of statin-based lipid-lowering therapy have failed to slow disease progression in established calcific AS [27,28,29], although ongoing trials are evaluating whether more intensive lipid-modifying strategies may alter this trajectory [30]. This apparent paradox suggests a stage-dependent relationship, in which lipid-mediated pathways may be more relevant during disease initiation and early progression but less influential once advanced calcification and fixed valvular obstruction have developed. These observations are consistent with the present findings, in which no statistically significant associations of AIP trajectory groups with major clinical outcomes or longitudinal hemodynamic recovery were detected after TAVR. After valve replacement, the prognostic importance of lipid-related metabolic exposure may be outweighed by procedural, anatomical, prosthesis-related, and comorbidity-related determinants of outcome.

### 4.4. Determinants of Outcomes After TAVR

A central observation of this study is that no statistically significant association between AIP trajectory groups and major adverse clinical outcomes was detected after TAVR. This finding was generally consistent across crude and multivariable analyses and across most prespecified subgroups.

This dissociation may be understood in the context of the pathophysiological changes induced by TAVR. By relieving fixed valvular obstruction, TAVR substantially alters the hemodynamic milieu and may shift the determinants of prognosis away from upstream metabolic factors toward postprocedural and patient-specific factors [5,6]. Outcomes after TAVR are influenced by multiple factors, including prosthesis performance, residual paravalvular regurgitation, conduction disturbances, vascular complications, anatomical features, frailty, and coexisting comorbidities [31,32,33,34,35]. In this context, lipid-related biomarkers may provide limited additional explanatory information beyond established clinical and procedural variables. Several secondary findings warrant cautious interpretation. Patients in Traj3 had higher observed rates of early disabling stroke and 1-year non-cardiovascular mortality, and a nominal interaction was observed among patients with moderate or greater baseline AR. However, these findings were based on relatively small numbers of events, arose in the context of multiple comparisons, and were not accompanied by a consistent overall association between trajectory groups and long-term outcomes. Moreover, some early events occurred before the longitudinal trajectory was fully characterized. These observations should therefore be considered exploratory and hypothesis-generating.

### 4.5. Valve Performance and Bioprosthetic Durability

No statistically significant association between AIP trajectories and longitudinal valve hemodynamics after TAVR was detected. Although several isolated between-group differences were observed at individual follow-up time points, LMM did not demonstrate significant trajectory-by-time interactions for peak aortic jet velocity, mean transvalvular gradient, LVEF, or LVMI. In addition, the incidence of BVF was low and showed no significant association with AIP trajectories. These observations suggest that mechanisms underlying bioprosthetic valve function and durability may differ from those driving native valve disease [7,31,36]. Although lipid-related mechanisms have been implicated in native valve calcification, their role in bioprosthetic valve degeneration remains less clearly established. Bioprosthetic degeneration is likely influenced by multiple mechanisms, including mechanical stress, leaflet material properties, valve expansion, leaflet thrombosis, residual regurgitation, inflammation, and procedural factors [31,36,37,38]. Therefore, the lack of a statistically significant association between AIP trajectories and valve hemodynamic recovery in the present study is biologically plausible and consistent with the multifactorial nature of bioprosthetic valve performance.

### 4.6. Clinical Implications

The relationship between lipid-related markers and outcomes after TAVR remains incompletely understood. Recent studies have reported that baseline AIP may be associated with adverse outcomes in patients with severe AS undergoing TAVR, suggesting that atherogenic dyslipidemia may reflect overall cardiovascular vulnerability before intervention [19,39]. The present findings should be interpreted in this context. Unlike prior studies that evaluated AIP as a single baseline exposure, this study modeled AIP as a longitudinal trajectory variable incorporating peri-procedural and follow-up measurements. This distinction is clinically and methodologically important. Baseline AIP may capture preprocedural metabolic risk and systemic cardiovascular vulnerability, whereas trajectory-based modeling reflects dynamic metabolic patterns over time, which may be influenced by postprocedural changes, medical therapy, nutritional status, survival selection, and follow-up intensity [19,20,21,39]. In the complementary baseline AIP analysis, baseline AIP was not significantly associated with MACCEs, stroke, heart failure rehospitalization, or BVF. Unexpectedly, higher baseline AIP was inversely associated with all-cause and cardiovascular mortality. Given the limited number of mortality events and the direction of these associations, these findings should be interpreted cautiously and should not be considered evidence of a protective effect of higher AIP.

Within this framework, the lack of a statistically significant association between AIP trajectory groups and the primary clinical outcome suggests that longitudinal AIP patterns may have limited prognostic relevance after TAVR in this cohort [19,36]. Rather than contradicting prior studies, these findings may indicate that clinical relevance of AIP is time-dependent and context-specific.

Furthermore, these findings are broadly aligned with randomized trials of lipid-lowering therapies in AS, which have not demonstrated a clear benefit in altering disease progression or post-intervention outcomes [28,39,40,41]. Importantly, these findings do not diminish the role of lipid management in overall cardiovascular risk reduction. The present study was not designed to evaluate the efficacy of lipid-lowering therapy, and detailed information regarding lipid-lowering regimens, treatment changes, and medication adherence was not consistently available. Therefore, the absence of a statistically significant association between AIP trajectories and the examined outcomes should not be interpreted as evidence that lipid-lowering therapy is unimportant after TAVR. These findings should not alter guideline-directed lipid-lowering therapy for established indications of atherosclerotic cardiovascular risk reduction. Rather, our findings may reflect the distinction between systemic atherosclerotic risk and the predominantly valvular, anatomical, and procedure-related determinants of outcomes after TAVR, underscoring the importance of interpreting the prognostic value of biomarkers within the relevant pathophysiological and clinical context [8,9,42,43,44,45].

### 4.7. Limitations

Several limitations should be considered when interpreting these findings. First, this was a retrospective analysis of data from a single-center registry, which may limit the generalizability of the findings to other populations, centers, and healthcare settings. Although consecutive patients were screened and multivariable adjustment was performed, residual confounding cannot be fully excluded. Second, selection related to longitudinal AIP availability is an important consideration. Trajectory classification required repeated AIP measurements, and patients who lacked sufficient longitudinal measurements could not be classified. Compared with patients included in the trajectory analysis, excluded patients had a generally higher-risk clinical profile. Therefore, the trajectory cohort represents a selected population, and the very low 30-day mortality observed in this cohort should not be interpreted as the overall early mortality of the source TAVR registry. Third, the number of long-term clinical events was limited, particularly for individual secondary endpoints, which may have reduced statistical power to detect modest associations between AIP trajectories and clinical outcomes. Several estimates therefore had wide confidence intervals, and lack of statistical significance should not be interpreted as definitive evidence of no association. Fourth, AIP trajectories were constructed using measurements obtained from within 7 days before TAVR through follow-up without a predefined landmark time point. This approach introduces temporal overlap between trajectory ascertainment and outcome occurrence. Therefore, the AIP trajectory groups should be interpreted as post hoc longitudinal metabolic patterns rather than baseline-defined prospective predictors, and the present analyses cannot establish causality. Landmark analyses using early 3- or 6-month windows were considered but were not performed because restricting trajectory construction to these periods substantially reduced the number of patients with sufficient repeated AIP measurements and left too few subsequent clinical events for stable multivariable analyses. Future studies with standardized serial sampling should evaluate landmark, time-dependent, or joint longitudinal-survival approaches. Fifth, trajectory modeling was based on AIP measurements obtained during routine clinical follow-up, with a minimum of two measurements required for inclusion. The number and timing of measurements therefore varied among patients and may not fully capture more complex or short-term metabolic changes. Sixth, variability in follow-up timing and incomplete availability of serial echocardiographic data may have introduced measurement heterogeneity, particularly during long-term follow-up when the number of available echocardiographic assessments decreased. Linear mixed-effects models incorporated all available observations without single-value imputation and rely on the assumption that missing observations are missing at random conditional on the observed data; informative missingness cannot be completely excluded. Finally, detailed information on lipid-lowering therapy, including treatment type and dose, initiation or discontinuation, treatment changes, and medication adherence, was not consistently available. Dietary patterns, nutritional status, and other metabolic modifiers were also not fully captured. Residual confounding by these factors therefore remains possible.

## 5. Conclusions

In patients undergoing TAVR, longitudinal AIP trajectory modeling identified distinct patterns of atherogenic dyslipidemia that corresponded to different cardiometabolic profiles. However, no statistically significant associations between AIP trajectory groups and major clinical outcomes or differential recovery of valve hemodynamics and left ventricular remodeling were detected. These findings suggest that longitudinal AIP patterns may have limited clinical relevance for differentiating post-TAVR outcomes in this study population, although interpretation is limited by temporal overlap in trajectory ascertainment, selection related to repeated AIP measurements, and the limited number of clinical events.

## Figures and Tables

**Figure 1:**
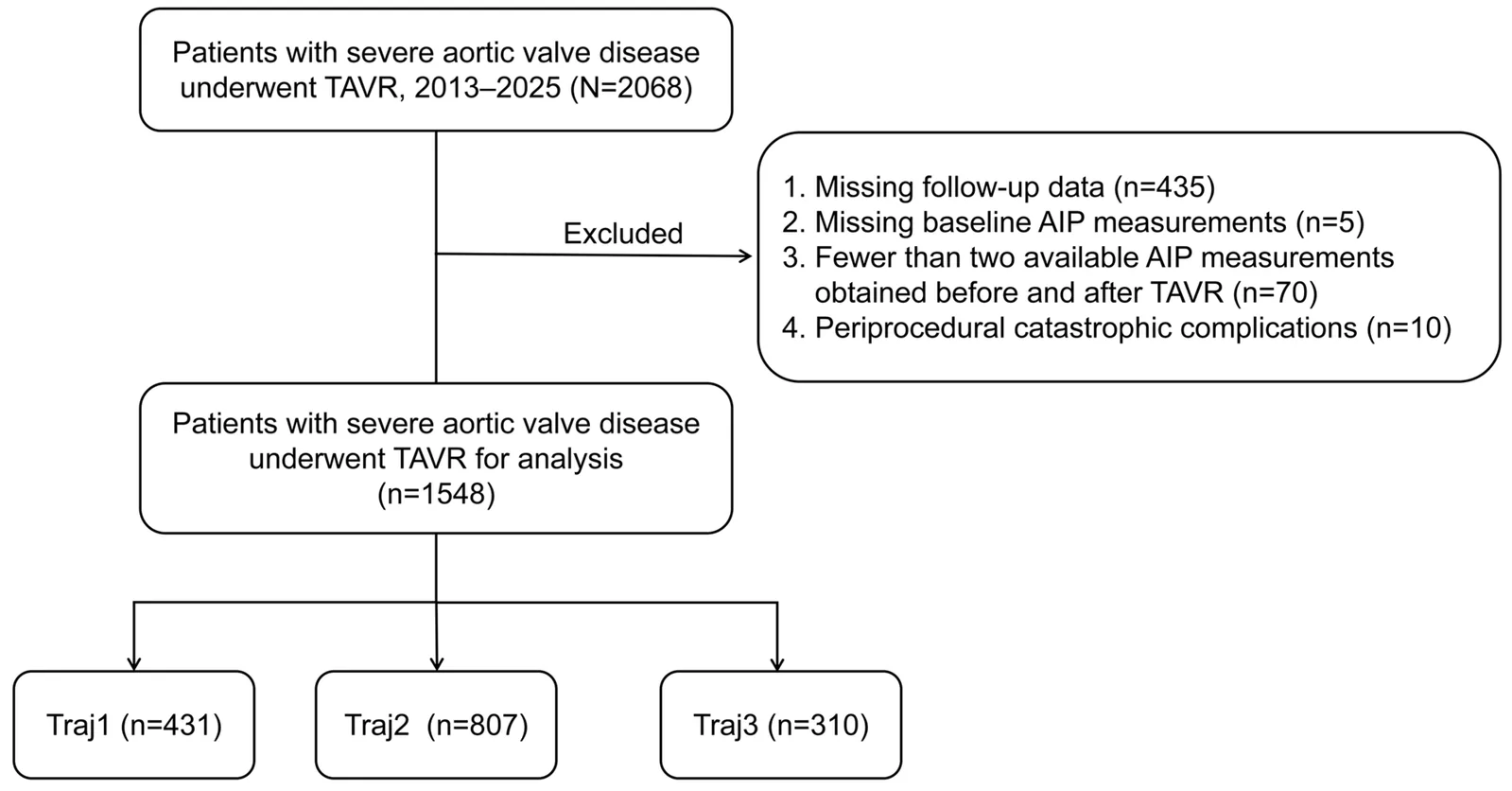
Study flow diagram. Abbreviations: AIP, atherogenic index of plasma; TAVR, transcatheter aortic valve replacement.

**Figure 2:**
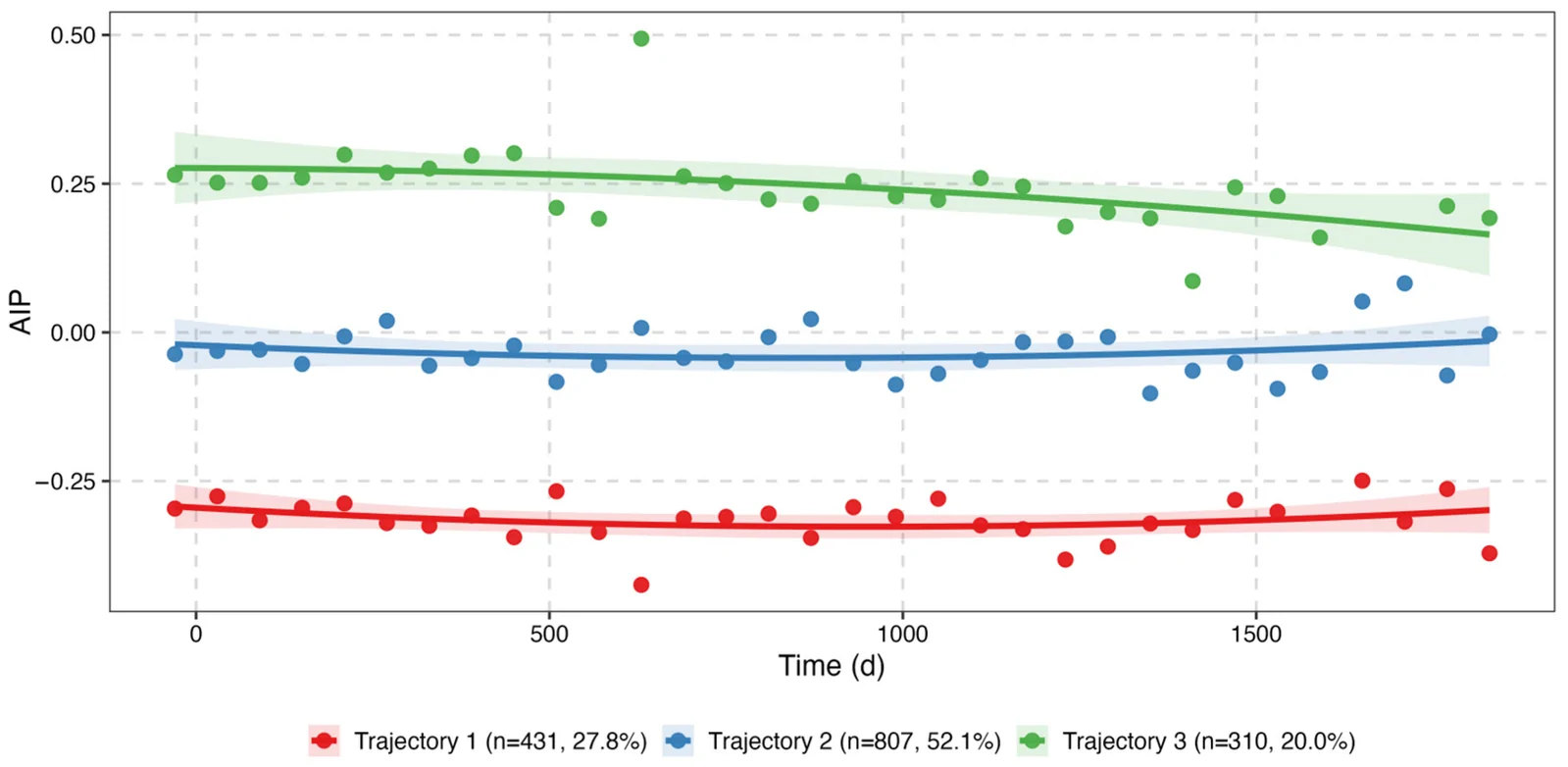
AIP trajectories identified by group-based trajectory modeling. Three distinct longitudinal AIP trajectories were identified. Traj1, Traj2, and Traj3 represent the low-level, intermediate-level, and high-level AIP trajectory groups, respectively. Abbreviations: AIP, atherogenic index of plasma.

**Figure 3:**
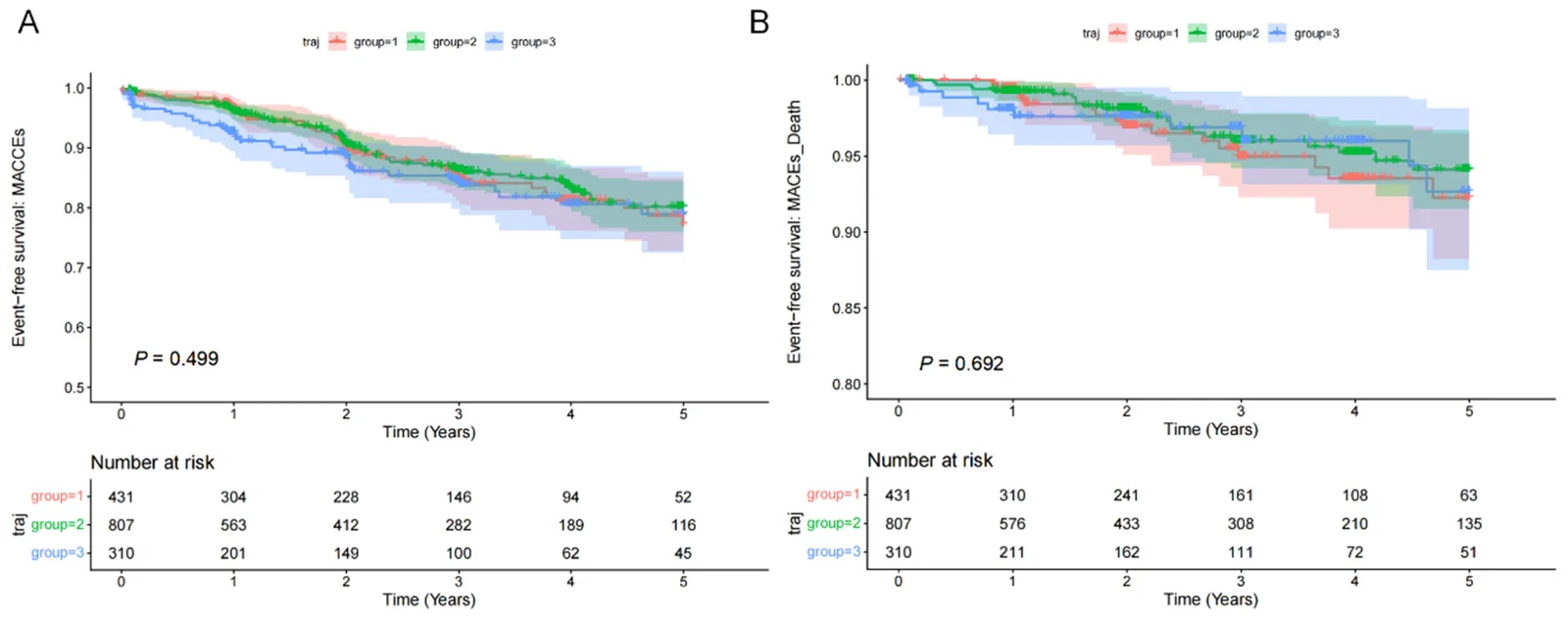
Kaplan–Meier curves for clinical outcomes according to AIP trajectories. Kaplan–Meier curves for 5-year MACCEs (**A**) and all-cause mortality (**B**) stratified by AIP trajectory groups. Traj1, Traj2, and Traj3 represent the low-level, intermediate-level, and high-level AIP trajectory groups, respectively. Abbreviations: AIP, atherogenic index of plasma; MACCEs, major adverse cardiovascular and cerebrovascular events.

**Figure 4:**
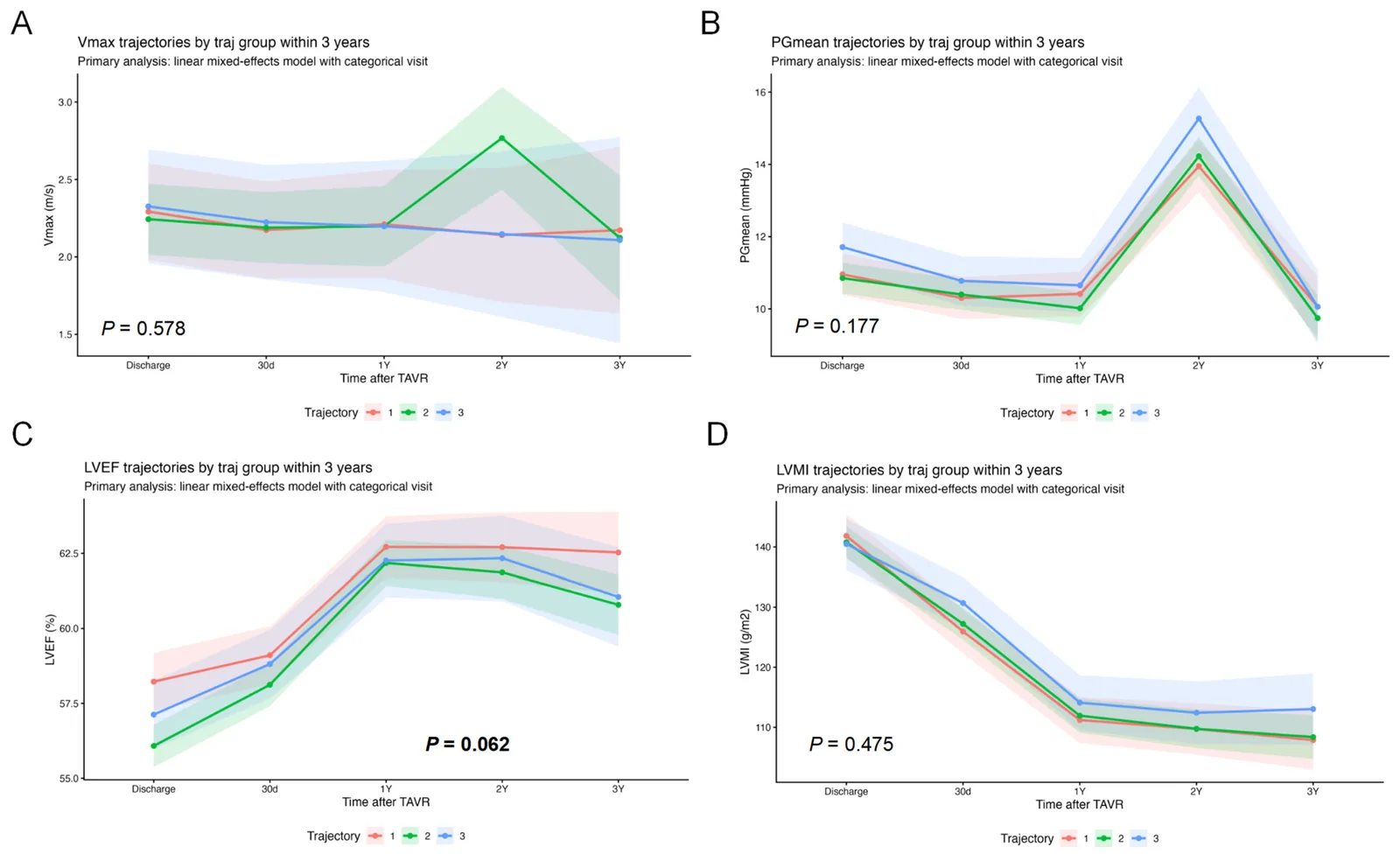
Longitudinal echocardiographic parameters according to AIP trajectories. Longitudinal changes in peak aortic jet velocity (Vmax) (**A**), mean transvalvular gradient (PGmean) (**B**), left ventricular ejection fraction (LVEF) (**C**), and left ventricular mass index (LVMI) (**D**) across AIP trajectory groups. Values are presented as estimated marginal means with 95% confidence intervals derived from linear mixed-effects models. Abbreviations: AIP, atherogenic index of plasma; CI, confidence interval; LVEF, left ventricular ejection fraction; LVMI, left ventricular mass index; PGmean, mean transvalvular gradient; Vmax, peak aortic jet velocity.

**Figure 5:**
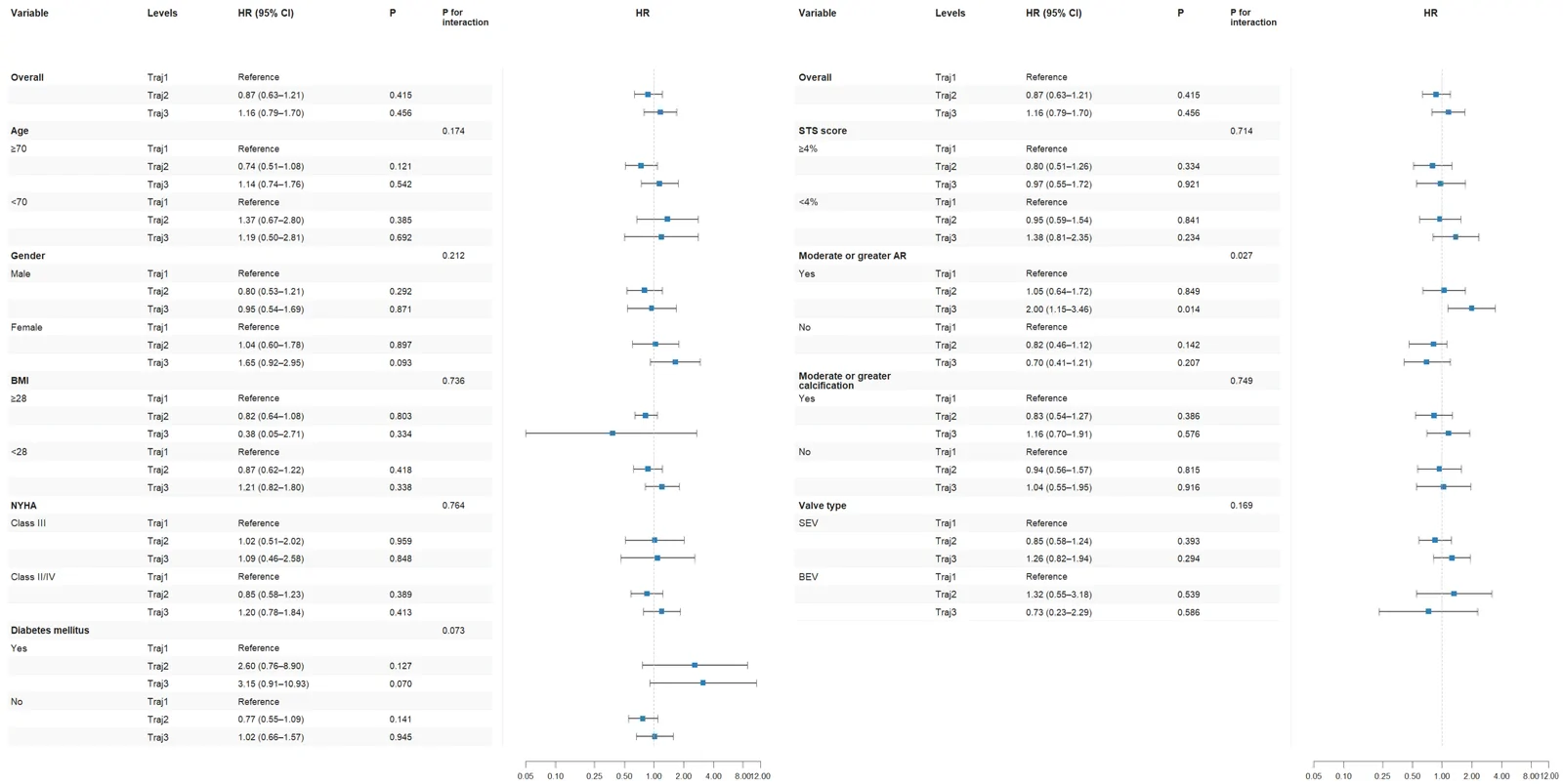
Subgroup analyses of the association between AIP trajectories and MACCEs. Hazard ratios and 95% confidence intervals for MACCEs across AIP trajectory groups are shown in predefined subgroups, using Traj1 as the reference. *p* values for interaction were calculated by including interaction terms between AIP trajectory groups and each subgroup variable in Cox proportional hazards models. Models were adjusted for the same covariates as in the primary analysis, except for the stratifying variable when applicable. Abbreviations: AIP, atherogenic index of plasma; AR, aortic regurgitation; BMI, body mass index; CI, confidence interval; HR, hazard ratio; LVEF, left ventricular ejection fraction; MACCEs, major adverse cardiovascular and cerebrovascular events; NYHA, New York Heart Association; SEV, self-expanding valve; STS, Society of Thoracic Surgeons; TAV, tricuspid aortic valve.

**Table 1:** Baseline characteristics according to AIP trajectory groups.

Variables	Overall (*n* = 1548)	Traj1 (*n* = 431)	Traj2 (*n* = 807)	Traj3 (*n* = 310)	*p* Value
Age, years	74 (69–79)	75 (70–80)	75 (70–79)	73 (68–78)	**<0.001**
Male	915 (59.1)	272 (63.1)	476 (59.0)	167 (53.9)	**0.041**
BMI, kg/m^2^	22.9 (20.5–25.1)	21.5 (19.5–23.6)	23.0 (20.7–25.2)	24.4 (22.4–26.5)	**<0.001**
BSA, m^2^	1.6 (1.5–1.8)	1.6 (1.5–1.7)	1.6 (1.5–1.8)	1.7 (1.6–1.8)	**<0.001**
STS-score, %	3.10 (1.87–5.43)	3.32 (2.06–5.90)	3.10 (1.83–5.34)	2.90 (1.75–4.79)	**0.015**
NYHA III/IV	956 (61.8)	278 (64.5)	490 (60.7)	188 (60.6)	0.386
Hypertension	897 (57.9)	233 (54.1)	467 (57.9)	197 (63.5)	**0.036**
Dyslipidemia	311 (20.1)	49 (11.4)	128 (15.9)	134 (43.2)	**<0.001**
Diabetes mellitus	274 (17.7)	60 (13.9)	130 (16.1)	84 (27.1)	**<0.001**
Chronic kidney disease	810 (52.3)	246 (57.1)	436 (54.0)	128 (41.3)	**<0.001**
COPD	222 (14.3)	76 (17.6)	115 (14.2)	31 (10.0)	**0.014**
AF	258 (16.7)	79 (18.3)	135 (16.7)	44 (14.2)	0.329
Coronary artery disease	149 (9.6)	31 (7.2)	78 (9.7)	40 (12.9)	**0.034**
Cerebrovascular events	94 (6.1)	26 (6.0)	44 (5.4)	24 (7.7)	0.357
Peripheral artery disease	106 (6.8)	23 (5.3)	54 (6.7)	29 (9.4)	0.099
Prior pacemaker implantation	45 (2.9)	23 (5.3)	18 (2.2)	4 (1.3)	**0.001**
Laboratory data					
Total cholesterol, mmol/L	3.89 (3.26–4.70)	3.82 (3.32–4.63)	3.88 (3.25–4.66)	4.01 (3.26–4.88)	0.195
Triglyceride, mmol/L	1.04 (0.79–1.40)	0.74 (0.61–0.91)	1.07 (0.86–1.31)	1.71 (1.32–2.16)	**<0.001**
LDL-C, mmol/L	1.99 (1.53–2.59)	1.81 (1.37–2.28)	2.01 (1.58–2.61)	2.23 (1.67–2.83)	**<0.001**
HDL-C, mmol/L	1.20 (1.01–1.41)	1.43 (1.28–1.70)	1.17 (1.01–1.33)	0.98 (0.85–1.12)	**<0.001**
AIP	−0.05 (−0.22–0.11)	−0.29 (−0.39-−0.19)	−0.03 (−0.14–0.07)	0.23 (0.13–0.36)	**<0.001**
Echocardiographic data					
Bicuspid aortic valve	652 (42.1)	183 (42.5)	331 (41.0)	138 (44.5)	0.737
Pure AS	740 (47.8)	197 (45.7)	376 (46.6)	167 (53.9)	0.055
Pure AR	325 (21.0)	95 (22.0)	178 (22.1)	52 (16.8)	0.125
Mixed aortic valve disease	483 (31.2)	139 (32.3)	253 (31.4)	91 (29.4)	0.697
Aortic valve area, cm^2^	0.73 (0.54–0.97)	0.71 (0.53–0.98)	0.73 (0.54–0.99)	0.74 (0.57–0.92)	0.384
Mean transvalvular gradient, mmHg	44 (29–58)	45 (30–64)	43 (24–57)	44 (34–59)	**0.028**
Peak aortic jet velocity, m/s	4.35 (3.57–4.97)	4.39 (3.63–5.16)	4.31 (3.29–4.89)	4.38 (3.98–4.97)	**0.018**
LVEF, %	60.3 (50.9–65.6)	60.5 (53.4–65.9)	59.8 (48.1–65.3)	61.0 (52.8–66.2)	**0.014**
LVMI, g/m^2^	140.4 (116.0–172.7)	144.5 (115.2–175.8)	138.3 (115.5–173.9)	141.4 (118.6–170.1)	0.462
Moderate or greater AR	806 (52.1)	237 (54.9)	426 (52.8)	143 (46.1)	0.050
Moderate or greater MR	279 (18.0)	86 (20.0)	145 (18.0)	48 (15.5)	0.305
Moderate or greater TR	176 (11.4)	76 (17.6)	79 (9.8)	21 (6.8)	**<0.001**
Computed tomography data					
Annulus area, mm^2^	471.5 (408.2–546.2)	471.5 (397.8–549.8)	475.4 (410.8–548.1)	465.5 (414.2–535.4)	0.586
Annulus perimeter, mm	78.4 (72.9–84.1)	78.1 (72.3–84.3)	78.7 (73.1–84.4)	77.8 (73.4–83.4)	0.485
Annular eccentricity, %	23.7 (18.2–30.0)	23.2 (17.9–29.6)	23.9 (18.5–30.1)	23.4 (17.6–30.1)	0.347
Sinotubular junction diameter, mm	31.1 (28.4–34.2)	31.3 (28.7–35.2)	31.1 (28.3–34.1)	30.9 (28.1–33.8)	0.213
Sinotubular junction height, mm	22.1 (19.6–25.0)	22.4 (19.7–25.2)	22.0 (19.5–25.0)	22.0 (19.6–24.4)	0.203
Maximum ascending aorta diameter, mm	40.8 (37.0–44.3)	41.2 (37.2–44.6)	40.6 (36.7–44.3)	40.8 (37.2–43.9)	0.393
Aortic angulation, degree	51 (44–58)	49 (42–57)	51 (45–59)	53 (46–60)	**<0.001**
Right coronary artery height, mm	16.8 (14.7–19.2)	16.7 (14.6–19.0)	16.8 (14.7–19.2)	16.8 (14.9–19.1)	0.809
Left main height, mm	14.1 (12.0–16.7)	14.4 (12.2–17.0)	14.2 (12.0–16.7)	13.7 (11.7–16.1)	0.087
Moderate or greater calcification of device landing zone	891 (58.8)	250 (58.8)	460 (58.2)	181 (60.5)	0.776

Values are presented as median (interquartile range) or *n* (%), unless otherwise indicated. Traj1, Traj2, and Traj3 represent the low-level, intermediate-level, and high-level AIP trajectory groups, respectively. A two-sided *p* value <0.05 was considered statistically significant; statistically significant *p* values are shown in bold. Abbreviations: AF, atrial fibrillation; AIP, atherogenic index of plasma; AR, aortic regurgitation; AS, aortic stenosis; BMI, body mass index; BSA, body surface area; COPD, chronic obstructive pulmonary disease; HDL-C, high-density lipoprotein cholesterol; LDL-C, low-density lipoprotein cholesterol; LVEF, left ventricular ejection fraction; LVMI, left ventricular mass index; MR, mitral regurgitation; NYHA, New York Heart Association; STS, Society of Thoracic Surgeons; TR, tricuspid regurgitation.

**Table 2:** Procedural characteristics and clinical outcomes after TAVR according to AIP trajectory groups.

Variables	Total (*n* = 1548)	Traj1 (*n* = 431)	Traj2 (*n* = 807)	Traj3 (*n* = 310)	*p* Value
Procedural data					
Transfemoral access	1379 (89.1)	391 (90.7)	712 (88.2)	276 (89.0)	0.408
Implanted valve type					0.225
Self-expanding valve	1142 (73.8)	328 (76.1)	584 (72.4)	230 (74.2)	
Balloon-expandable valve	218 (14.1)	63 (14.6)	111 (13.8)	44 (14.2)	
Other valve types	188 (12.1)	40 (9.3)	112 (13.9)	36 (11.6)	
Annular oversizing ratio, %	6.2 (1.1–12.1)	6.5 (1.5–13.1)	6.1 (1.2–12.0)	7.1 (0.6–12.0)	0.621
Pre-dilatation,	1055 (68.2)	288 (66.8)	541 (67.0)	226 (72.9)	0.133
Post-dilatation	937 (60.5)	269 (62.4)	478 (59.2)	190 (61.3)	0.526
Valve repositioning	565 (36.5)	160 (37.1)	292 (36.2)	113 (36.4)	0.948
In-hospital/discharge outcomes					
Stroke	40 (2.6)	12 (2.8)	15 (1.9)	13 (4.2)	0.085
Disabling stroke	10 (0.6)	3 (0.7)	2 (0.2)	5 (1.6)	**0.026**
Non-disabling stroke	30 (1.9)	9 (2.1)	13 (1.6)	8 (2.6)	0.085
Myocardial infarction	4 (0.3)	2 (0.5)	1 (0.1)	1 (0.3)	0.420
Severe bleeding	88 (5.7)	20 (4.6)	55 (6.8)	13 (4.2)	0.135
Major vascular complication	15 (1.0)	5 (1.2)	8 (1.0)	2 (0.6)	0.837
New-onset AF	128 (8.3)	40 (9.3)	61 (7.6)	27 (8.7)	0.548
New permanent pacemaker	128 (8.3)	38 (8.8)	63 (7.8)	27 (8.7)	0.793
30-day clinical outcomes					
All-cause mortality	2 (0.1)	2 (0.5)	0 (0.0)	0 (0.0)	-
Cardiovascular mortality	2 (0.1)	2 (0.5)	0 (0.0)	0 (0.0)	-
Non-cardiovascular mortality	0 (0.0)	0 (0.0)	0 (0.0)	0 (0.0)	-
Stroke	43 (2.8)	12 (2.8)	16 (2.0)	15 (4.8)	**0.034**
Disabling stroke	11 (0.7)	3 (0.7)	2 (0.2)	6 (1.9)	**0.011**
Non-disabling stroke	32 (2.1)	9 (2.1)	14 (1.7)	9 (2.9)	0.470
Myocardial infarction	4 (0.3)	2 (0.5)	1 (0.1)	1 (0.3)	0.420
Heart failure rehospitalization	3 (0.2)	0 (0.0)	3 (0.4)	0 (0.0)	-
1-year clinical outcomes					
All-cause mortality	17 (1.1)	4 (0.9)	6 (0.7)	7 (2.3)	0.109
Cardiovascular mortality	9 (0.6)	3 (0.7)	4 (0.5)	2 (0.6)	0.825
Non-cardiovascular mortality	8 (0.5)	1 (0.2)	2 (0.2)	5 (1.6)	**0.019**
Stroke	57 (3.7)	15 (3.5)	24 (3.0)	18 (5.8)	0.076
Disabling stroke	14 (0.9)	4 (0.9)	3 (0.4)	7 (2.3)	**0.013**
Non-disabling stroke	43 (2.8)	11 (2.6)	21 (2.6)	11 (3.5)	0.671
Myocardial infarction	6 (0.4)	3 (0.7)	1 (0.1)	2 (0.6)	0.164
Heart failure re-hospitalization	18 (1.2)	3 (0.7)	12 (1.5)	3 (1.0)	0.442
BVF	2 (0.1)	1 (0.2)	1 (0.1)	0 (0.0)	-

Values are presented as median (interquartile range) or *n* (%), unless otherwise indicated. Traj1, Traj2, and Traj3 represent the low-level, intermediate-level, and high-level AIP trajectory groups, respectively. A two-sided *p* value <0.05 was considered statistically significant; statistically significant *p* values are shown in bold. *p* values were not calculated for variables with sparse or zero events across groups. Abbreviations: AF, atrial fibrillation; AIP, atherogenic index of plasma; BVF, bioprosthetic valve failure; TAVR, transcatheter aortic valve replacement.

**Table 3:** Associations of baseline AIP and AIP trajectories with clinical outcomes in the trajectory-analysis cohort.

	Crude Model		Model I		Model II	
	Crude HR (95% CI)	*p* Value	Adjusted HR (95% CI)	*p* Value	Adjusted HR (95% CI)	*p* Value
MACCEs						
AIP	0.95 (0.54–1.68)	0.863	0.89 (0.50–1.58)	0.691	0.84 (0.47–1.49)	0.557
Traj1	Reference		Reference		Reference	
Traj2	0.87 (0.63–1.21)	0.415	0.86 (0.62–1.19)	0.356	0.83 (0.60–1.16)	0.277
Traj3	1.16 (0.79–1.70)	0.456	1.12 (0.76–1.65)	0.566	1.09 (0.74–1.61)	0.663
All-cause mortality						
AIP	0.33 (0.11–0.97)	**0.043**	0.35 (0.12–1.03)	0.058	0.34 (0.11–1.02)	0.055
Traj1	Reference		Reference		Reference	
Traj2	0.70 (0.40–1.22)	0.207	0.71 (0.40–1.26)	0.231	0.71 (0.41–1.26)	0.242
Traj3	0.70 (0.34–1.46)	0.340	0.73 (0.35–1.53)	0.408	0.75 (0.35–1.57)	0.442
Cardiovascular mortality						
AIP	0.11 (0.02–0.72)	**0.021**	0.10 (0.02–0.62)	**0.014**	0.10 (0.01–0.62)	**0.014**
Traj1	Reference		Reference		Reference	
Traj2	0.58 (0.24–1.43)	0.235	0.56 (0.23–1.37)	0.205	0.58 (0.23–1.43)	0.239
Traj3	0.46 (0.12–1.70)	0.244	0.40 (0.11–1.50)	0.174	0.40 (0.10–1.50)	0.173
Stroke						
AIP	1.08 (0.36–3.22)	0.896	1.02 (0.34–3.08)	0.973	1.08 (0.36–3.27)	0.885
Traj1	Reference		Reference		Reference	
Traj2	0.57 (0.30–1.06)	0.074	0.56 (0.30–1.04)	0.067	0.57 (0.30–1.06)	0.075
Traj3	1.00 (0.50–1.99)	0.989	0.96 (0.48–1.93)	0.912	1.04 (0.51–2.10)	0.923
Heart failure rehospitalization						
AIP	1.10 (0.36–3.40)	0.868	1.13 (0.36–3.52)	0.833	0.93 (0.30–2.88)	0.894
Traj1	Reference		Reference		Reference	
Traj2	1.41 (0.71–2.81)	0.331	1.42 (0.71–2.84)	0.321	1.32 (0.66–2.65)	0.432
Traj3	1.25 (0.53–2.95)	0.608	1.27 (0.54–3.01)	0.584	1.11 (0.46–2.66)	0.816
BVF						
AIP	2.10 (0.70–6.35)	0.187	1.55 (0.51–4.74)	0.440	1.43 (0.46–4.42)	0.535
Traj1	Reference		Reference		Reference	
Traj2	1.02 (0.51–2.04)	0.949	0.96 (0.48–1.91)	0.902	0.90 (0.45–1.80)	0.758
Traj3	1.57 (0.73–3.40)	0.252	1.35 (0.62–2.93)	0.451	1.27 (0.58–2.80)	0.550

HRs were estimated using Cox proportional hazards models. Baseline AIP and AIP trajectory group were evaluated in separate Cox proportional hazards models among the 1548 patients included in the trajectory analysis. Baseline AIP was analyzed as a continuous variable per 1-unit increase, and Traj1 was used as the reference group for trajectory analyses. Model I was adjusted for age and sex. Model II was additionally adjusted for NYHA class III/IV, hypertension, and diabetes mellitus. A two-sided *p* value <0.05 was considered statistically significant; statistically significant *p* values are shown in bold. Abbreviations: AIP, atherogenic index of plasma; BVF, bioprosthetic valve failure; CI, confidence interval; HR, hazard ratio; MACCEs, major adverse cardiovascular and cerebrovascular events; NYHA, New York Heart Association.

## Data Availability

The data underlying this article are not publicly available due to patient privacy and institutional restrictions. Deidentified data may be made available from the corresponding author upon reasonable request and with permission from the Second Affiliated Hospital of Zhejiang University School of Medicine.

## Supplementary Materials

The following supporting information can be downloaded at https://www.mdpi.com/article/10.3390/jcm15197448/s1. Table S1: Group-based trajectory model fit indices; Table S2: Baseline characteristics of patients included in and excluded from the AIP trajectory analysis.; Table S3: Baseline characteristics among patients with available baseline AIP according to inclusion in the AIP trajectory analysis. Table S4: Associations between baseline AIP and clinical outcomes in the broader baseline-AIP cohort; Table S5: Echocardiographic parameters at each follow-up time point according to AIP trajectory groups.

## Abbreviations

AF, atrial fibrillation; AIC, Akaike Information Criterion; AIP, atherogenic index of plasma; AR, aortic regurgitation; AS, aortic stenosis; AVD, aortic valve disease; BIC, Bayesian Information Criterion; BMI, body mass index; BSA, body surface area; BVF, bioprosthetic valve failure; CI, confidence interval; COPD, chronic obstructive pulmonary disease; GBTM, group-based trajectory modeling; HDL-C, high-density lipoprotein cholesterol; HR, hazard ratio; IQR, interquartile range; LDL-C, low-density lipoprotein cholesterol; LMM, linear mixed-effects model; LVEF, left ventricular ejection fraction; LVMI, left ventricular mass index; MACCEs, major adverse cardiovascular and cerebrovascular events; NYHA, New York Heart Association; PGmean, mean transvalvular gradient; PPM, prosthesis–patient mismatch; PVR, paravalvular regurgitation; SD, standard deviation; SEV, self-expanding valve; STS, Society of Thoracic Surgeons; TAV, tricuspid aortic valve; TAVR, transcatheter aortic valve replacement; TORCH, Transcatheter Aortic Valve Replacement Single-Center Registry in the Chinese Population; Vmax, peak aortic jet velocity.
