# Supplementary material for: Longitudinal Trajectories of the Atherogenic Index of Plasma and Their Associations with Clinical Outcomes After Transcatheter Aortic Valve Replacement
Source: J Clin Med. 2026 Sep 24;15(19):7448. doi: 10.3390/jcm15197448 (PMC13665838; doi:10.3390/jcm15197448)
Supplement: Supplementary file 1 [file jcm-15-07448-s001.zip › jcm-4516425-supplementary.pdf]

Supplementary data

Supplementary Tables

Supplementary Table S1. Group-based trajectory model fit indices.

Number of groups	BIC (N=1548)	AIC	Log-likelihood	Entropy	Class 1 (%)	Class 2 (%)	Class 3 (%)	Class 4 (%)	Class 5 (%)
2	942.19	963.56	971.56	0.783	62.2	37.8			
3	1395.67	1427.74	1439.74	0.780	27.8	52.1	20.0		
4	1560.72	1603.48	1619.48	0.799	20.5	48.7	27.1	3.8	
5	1679.51	1732.95	1752.95	0.756	12.8	38.3	31.8	15.8	1.3

Abbreviations: AIC, Akaike Information Criterion; BIC, Bayesian Information Criterion.

Supplementary Table S2. Baseline characteristics of patients included in and excluded from the AIP trajectory analysis.

Variables	Overall (n=2068)	Included group (n=1548)	Excluded group (n=520)	*P* value
Age, years	75 (70–80)	74 (69–79)	77 (72–82)	**<0.001**
Male	1230 (59.5)	915 (59.1)	315 (60.6)	0.555
BMI, kg/m^2	22.7 (20.3–25.0)	22.9 (20.5–25.1)	22.2 (19.8–24.7)	**0.001**
BSA, m^2	1.6 (1.5–1.8)	1.6 (1.5–1.8)	1.6 (1.5–1.7)	**0.008**
STS-score, %	3.66 (2.07–6.45)	3.10 (1.87–5.43)	5.60 (3.53–9.46)	**<0.001**
NYHA III/IV	1378 (66.6)	956 (61.8)	422 (81.2)	**<0.001**
Hypertension	1178 (57.0)	897 (57.9)	281 (54.0)	0.119
Dyslipidemia	402 (19.4)	311 (20.1)	91 (17.5)	0.197
Diabetes mellitus	386 (18.7)	274 (17.7)	112 (21.5)	0.052
Chronic kidney disease	1176 (56.9)	810 (52.3)	366 (70.4)	**<0.001**
COPD	322 (15.6)	222 (14.3)	100 (19.2)	**0.008**
AF	384 (18.6)	258 (16.7)	126 (24.2)	**<0.001**
Coronary artery disease	215 (10.4)	149 (9.6)	66 (12.7)	**0.047**
Cerebrovascular events	127 (6.1)	94 (6.1)	33 (6.4)	0.822
Peripheral artery disease	198 (9.6)	106 (6.8)	92 (17.7)	**<0.001**
Prior pacemaker implantation	64 (3.1)	45 (2.9)	19 (3.6)	0.395
Laboratory data				
Total cholesterol, mmol/L*	3.88 (3.25–4.67)	3.89 (3.26–4.70)	3.74 (3.17–4.48)	0.100
Triglyceride, mmol/L*	1.03 (0.78–1.40)	1.04 (0.79–1.40)	0.98 (0.76–1.38)	**0.117**
LDL-C, mmol/L*	1.97 (1.52–2.57)	1.99 (1.53–2.59)	1.87 (1.45–2.57)	**0.041**
HDL-C, mmol/L*	1.19 (1.00–1.41)	1.20 (1.01–1.41)	1.16 (0.97–1.41)	0.072
AIP	−0.05 (−0.21–0.11)	−0.05 (−0.22–0.11)	−0.06 (−0.22–0.11)	0.981
Echocardiographic data				
Bicuspid aortic valve	846 (41.4)	652 (42.1)	194 (37.6)	**0.045**
Pure AS	1003 (48.5)	740 (47.8)	263 (50.6)	0.274
Pure AR	394 (19.1)	325 (21.0)	69 (13.3)	**<0.001**
Mixed aortic valve disease	671 (32.4)	483 (31.2)	188 (36.2)	**0.037**
Aortic valve area, cm^2	0.70 (0.52–0.92)	0.73 (0.54–0.97)	0.63 (0.48–0.97)	**<0.001**
Mean transvalvular gradient, mmHg	44 (32–60)	44 (29–58)	46 (36–61)	**0.003**
Peak aortic jet velocity, m/s	4.38 (3.77–5.01)	4.35 (3.57–4.97)	4.50 (4.03–5.10)	**<0.001**
LVEF, %	59.7 (49.2–65.3)	60.3 (50.9–65.6)	57.3 (45.1–63.4)	**<0.001**
LVMI, g/m^2	143.04 (118.6–177.0)	140.4 (116.0–172.7)	150.9 (126.4–190.5)	**<0.001**
Computed tomography data				
Annulus area, mm^2	469.0 (406.6–543.7)	471.5 (408.2–546.2)	461.4 (404.6–536.9)	0.217
Annulus perimeter, mm	78.3 (72.8–84.1)	78.4 (72.9–84.1)	77.6 (72.8–84.0)	0.335
Annular eccentricity, %	19.53 (8.37–27.23)	23.7 (18.2–30.0)	6.26 (4.75–7.78)	**<0.001**
Sinotubular junction diameter, mm	31.1 (28.3–34.3)	31.1 (28.4–34.2)	31.0 (28.0–34.4)	0.513
Sinotubular junction height, mm	22.1 (19.7–25.1)	22.1 (19.6–25.0)	22.2 (20.1–25.0)	0.095
Maximum ascending aorta diameter, mm	40.6 (36.8–44.4)	40.8 (37.0–44.3)	40.1 (36.6–44.6)	0.550
Aortic angulation, degree	51 (45–58)	51 (44–58)	52 (45–59)	0.244
Right coronary artery height, mm	16.8 (14.8–19.1)	16.8 (14.7–19.2)	16.7 (14.8–19.1)	0.927
Left main height, mm	14.1 (12.0–16.7)	14.1 (12.0–16.7)	14.2 (11.9–16.7)	0.848
Moderate or greater calcification of device landing zone	1231 (61.7)	891 (58.8)	350 (73.1)	**<0.001**

Values are presented as median (interquartile range) or n (%), unless otherwise indicated. A two-sided P value <0.05 was considered statistically significant. *Lipid

parameters were unavailable for 266 patients in the excluded group. Abbreviations: AF, atrial fibrillation; AIP, atherogenic index of plasma; AR, aortic regurgitation; AS, aortic stenosis; BMI, body mass index; BSA, body surface area; COPD, chronic obstructive pulmonary disease; HDL-C, high-density lipoprotein cholesterol; LDL-C, low-density lipoprotein cholesterol; LVEF, left ventricular ejection fraction; LVMI, left ventricular mass index; MR, mitral regurgitation; NYHA, New York Heart Association; STS, Society of Thoracic Surgeons; TR, tricuspid regurgitation.

Supplementary Table S3. Baseline characteristics among patients with available baseline AIP according to inclusion in the AIP trajectory analysis.

Variables	With baseline AIP (n=1628)	With AIP trajectory (n=1548)	With baseline AIP excluded (n=80)	*P* value
Age, years	74 (69–79)	74 (69–79)	75 (70–80)	0.513
Male	957 (58.8)	915 (59.1)	42 (52.5)	0.242
BMI, kg/m^2	22.9 (20.5–25.1)	22.9 (20.5–25.1)	22.3 (19.1–25.3)	0.133
BSA, m^2	1.6 (1.5–1.8)	1.6 (1.5–1.8)	1.6 (1.5–1.7)	**0.013**
STS-score, %	3.16 (1.88–5.57)	3.10 (1.87–5.43)	4.64 (2.34–8.24)	**<0.001**
NYHA III/IV	1006 (61.8)	956 (61.8)	50 (62.5)	0.894
Hypertension	938 (57.6)	897 (58.0)	41 (51.2)	0.237
Dyslipidemia	320 (19.7)	311 (20.1)	9 (11.2)	0.052
Diabetes mellitus	287 (17.6)	274 (17.7)	13 (16.2)	0.740
Chronic kidney disease	859 (52.8)	810 (52.3)	49 (61.2)	0.119
COPD	233 (14.3)	222 (14.3)	11 (13.8)	0.883
AF	280 (17.0)	258 (16.7)	22 (27.5)	**0.012**
Coronary artery disease	156 (9.6)	149 (9.6)	7 (8.8)	0.795
Cerebrovascular events	99 (6.1)	94 (6.1)	5 (6.2)	1.000
Peripheral artery disease	113 (6.9)	106 (6.8)	7 (8.8)	0.514
Prior pacemaker implantation	50 (3.1)	45 (2.9)	5 (6.2)	0.175
Laboratory data				
Total cholesterol, mmol/L	3.88 (3.26–4.69)	3.89 (3.27–4.69)	3.81 (3.24–4.57)	0.497
Triglyceride, mmol/L	1.04 (0.79–1.40)	1.04 (0.79–1.40)	1.03 (0.76–1.38)	0.475
LDL-C, mmol/L	1.98 (1.53–2.58)	1.99 (1.53–2.58)	1.89 (1.55–2.44)	0.392
HDL-C, mmol/L	1.20 (1.01–1.41)	1.20 (1.01–1.41)	1.21 (0.99–1.46)	0.664
AIP	−0.05 (−0.22–0.11)	−0.05 (−0.21–0.11)	−0.04 (−0.28–0.09)	0.543
Echocardiographic data				
Bicuspid aortic valve	679 (42.2)	652 (42.1)	27 (33.8)	0.118
Pure AS	776 (47.7)	740 (47.5)	36 (45.0)	0.624
Pure AR	343 (21.1)	325 (21.0)	18 (22.5)	0.748
Mixed aortic valve disease	505 (31.0)	483 (31.2)	22 (27.5)	0.485
Aortic valve area, cm^2	0.73 (0.54–0.97)	0.73 (0.54–0.97)	0.69 (0.55–0.84)	0.185
Mean transvalvular gradient, mmHg	43 (29–58)	44 (29–58)	41 (28–50)	0.080
Peak aortic jet velocity, m/s	4.33 (3.57–4.96)	4.35 (3.57–4.97)	4.16 (3.52–4.61)	0.052
LVEF, %	60.2 (50.6–65.6)	60.3 (50.9–65.6)	56.6 (45.4–62.7)	**0.012**
LVMI, g/m^2	140.4 (116.0–173.1)	140.4 (116.0–172.7)	139.1 (121.6–190.6)	0.395
Computed tomography data				
Annulus area, mm^2	470.3 (406.4–546.1)	471.5 (408.2–546.2)	453.4 (387.8–540.9)	0.264
Annulus perimeter, mm	78.4 (72.8–84.2)	78.4 (72.9–84.1)	77.2 (71.0–84.6)	0.435
Annular eccentricity, %	23.7 (18.2–30.2)	23.7 (18.2–30.0)	25.0 (21.1–31.8)	**0.031**
Sinotubular junction diameter, mm	31.1 (28.3–34.3)	31.1 (28.4–34.2)	31.2 (27.8–34.8)	0.796
Sinotubular junction height, mm	22.1 (19.6–25.0)	22.1 (19.6–25.0)	22.0 (19.9–25.5)	0.586
Maximum ascending aorta diameter, mm	40.8 (36.9–44.3)	40.8 (37.0–44.3)	41.4 (36.5–44.7)	0.790
Aortic angulation, degree	51 (44–58)	51 (44–58)	50 (43–57.5)	0.458
Right coronary artery height, mm	16.8 (14.8–19.2)	16.8 (14.7–19.2)	17.2 (15.4–20.0)	0.093
Left main height, mm	14.0 (12.0–16.7)	14.1 (12.0–16.7)	13.2 (11.2–15.6)	**0.035**
Moderate or greater calcification of device landing zone	932 (58.4)	891 (58.8)	41 (51.2)	0.181

Values are presented as median (interquartile range) or n (%), unless otherwise

indicated. A two-sided P value <0.05 was considered statistically significant. Abbreviations: AF, atrial fibrillation; AIP, atherogenic index of plasma; AR, aortic regurgitation; AS, aortic stenosis; BMI, body mass index; BSA, body surface area; COPD, chronic obstructive pulmonary disease; HDL-C, high-density lipoprotein cholesterol; LDL-C, low-density lipoprotein cholesterol; LVEF, left ventricular ejection fraction; LVMI, left ventricular mass index; MR, mitral regurgitation; NYHA, New York Heart Association; STS, Society of Thoracic Surgeons; TR, tricuspid regurgitation.

Supplementary Table S4. Associations Between Baseline AIP and Clinical Outcomes in the Broader Baseline-AIP Cohort.

	**Crude Model**		**Model I**		**Model II**	
	**Crude HR (95% CI)**	***P* value**	**Adjusted HR (95% CI)**	***P* value**	**Adjusted HR (95% CI)**	***P* value**
			MACCEs			
AIP	0.89 (0.52–1.50)	0.652	0.83 (0.48–1.41)	0.481	0.75 (0.43–1.30)	0.309
Low-AIP	Reference		Reference		Reference	
Medium-AIP	0.86 (0.63–1.17)	0.327	0.85 (0.62–1.16)	0.315	0.85 (0.62–1.16)	0.294
High-AIP	0.91 (0.67–1.24)	0.547	0.89 (0.65–1.21)	0.443	0.84 (0.61–1.16)	0.281
			All-cause mortality			
AIP	0.26 (0.10–0.66)	**0.005**	0.28 (0.11–0.70)	**0.007**	0.30 (0.11–0.80)	**0.016**
Low-AIP	Reference		Reference		Reference	
Medium-AIP	0.67 (0.41–1.11)	0.119	0.68 (0.41–1.11)	0.123	0.70 (0.42–1.15)	0.155
High-AIP	0.50 (0.29–0.87)	**0.014**	0.51 (0.29–0.89)	**0.018**	0.56 (0.31–0.99)	**0.048**
			Cardiovascular mortality			
AIP	0.08 (0.02–0.35)	**<0.001**	0.07 (0.02–0.33)	**<0.001**	0.08 (0.02–0.37)	**0.001**
Low-AIP	Reference		Reference		Reference	
Medium-AIP	0.44 (0.20–0.97)	**0.043**	0.44 (0.20–0.96)	**0.038**	0.46 (0.21–1.01)	0.052
High-AIP	0.31 (0.12–0.76)	**0.011**	0.29 (0.12–0.72)	**0.008**	0.33 (0.13–0.86)	**0.023**
			Stroke			
AIP	1.43 (0.52–3.90)	0.485	1.34 (0.49–3.69)	0.571	1.19 (0.41–3.43)	0.750
Low-AIP	Reference		Reference		Reference	
Medium-AIP	0.98 (0.51–1.88)	0.950	0.97 (0.51–1.87)	0.934	0.98 (0.51–1.89)	0.955
High-AIP	1.44 (0.79–2.64)	0.231	1.41 (0.77–2.58)	0.270	1.37 (0.73–2.57)	0.332
			Heart failure rehospitalization			
AIP	1.05 (0.35–3.09)	0.934	1.08 (0.36–3.21)	0.893	1.00 (0.32–3.07)	0.996
Low-AIP	Reference		Reference		Reference	
Medium-AIP	0.87 (0.44–1.71)	0.692	0.87 (0.45–1.71)	0.693	0.85 (0.43–1.68)	0.645
High-AIP	1.17 (0.62–2.19)	0.635	1.18 (0.63–2.22)	0.609	1.13 (0.58–2.17)	0.725
			BVF			
AIP	2.34 (0.81–6.75)	0.116	1.60 (0.55–4.67)	0.386	1.35 (0.44–4.12)	0.600
Low-AIP	Reference		Reference		Reference	
Medium-AIP	1.15 (0.58–2.25)	0.693	1.21 (0.61–2.38)	0.583	1.13 (0.45–1.82)	0.723
High-AIP	1.23 (0.63–2.37)	0.542	1.10 (0.57–2.13)	0.778	0.94 (0.47–1.87)	0.860

HRs were estimated using Cox proportional hazards models. This analysis included

1,628 patients with available baseline-AIP measurements, irrespective of eligibility for longitudinal trajectory classification. Baseline AIP was analyzed as a continuous variable per 1-unit increase, and Low-AIP was used as the reference group for the tertiles. Model I was adjusted for age and sex. Model II was additionally adjusted for NYHA class III/IV, hypertension, and diabetes mellitus. A two-sided P value <0.05 was considered statistically significant. Abbreviations: AIP, atherogenic index of plasma; BVF, bioprosthetic valve failure; CI, confidence interval; HR, hazard ratio; MACCEs, major adverse cardiovascular and cerebrovascular events; NYHA, New York Heart Association.

Supplementary Table S5. Echocardiographic parameters at each follow-up time point according to AIP trajectory groups.

At discharge	Total (n=1545)	Traj1 (n=430)	Traj2 (n=806)	Traj3 (n=309)	*P* value
Aortic valve area, cm^2	1.69 (1.39–2.01)	1.71 (1.41–2.04)	1.68 (1.39–2.03)	1.71 (1.38–1.95)	0.554
Peak aortic jet velocity, m/s	2.21 (1.88–2.59)	2.21 (1.87–2.63)	2.20 (1.88–2.54)	2.27 (1.93–2.64)	0.148
Mean transvalvular gradient, mmHg	10 (7–14)	10 (7–14)	10(7–13)	10(7–14)	0.146
LVEF, %	59.8 (50.5–65.0)	60.9 (53.2–65.8)	59.45 (48.2–64.7)	59.6 (51.6–64.8)	**0.012**
LVMI, g/m^2	134.1 (111.1–163.6)	134.8 (112.1–165.4)	132.7 (110.0–163.7)	136.6 (115.1–162.0)	0.725
Moderate or greater AR	6 (0.4)	2 (0.5)	2 (0.2)	2 (0.6)	0.562
Moderate or greater MR	143 (9.3)	41 (9.5)	76 (9.4)	26 (8.4)	0.848
Moderate or greater TR	110 (7.1)	47 (10.9)	48 (6.0)	15 (4.8)	**0.001**
**At 30 days**	**Total (n=1529)**	**Traj1 (n=426)**	**Traj2 (n=797)**	**Traj3 (n=306)**	***P* value**
Aortic valve area, cm^2	1.67 (1.41–1.98)	1.66 (1.41–1.97)	1.68 (1.40–2.01)	1.66 (1.44–1.94)	0.861
Peak aortic jet velocity, m/s	2.17 (1.84–2.50)	2.18 (1.81–2.52)	2.15 (1.83–2.50)	2.21 (1.91–2.49)	0.273
Mean transvalvular gradient, mmHg	9 (7–13)	9 (6–13)	9 (7–13)	10 (7–13)	0.229
LVEF, %	60.8 (54.6–65.8)	61.1 (55.5–66.2)	60.5 (54.1–65.6)	60.8 (55.2–65.4)	0.198
LVMI, g/m^2	122.8 (101.5–146.4)	120.4 (99.1–147.1)	123.5 (101.3–145.6)	125.4 (104.8–149.0)	0.406
Moderate or greater PPM	319 (20.9)	81 (19.0)	166 (20.8)	72 (23.5)	0.333

Moderate or greater AR	6 (0.4)	2 (0.5)	2 (0.2)	2 (0.6)	0.563
Moderate or greater MR	89 (5.8)	32 (7.5)	41 (5.1)	16 (5.2)	0.214
Moderate or greater TR	98 (6.4)	46 (10.8)	39 (4.9)	13 (4.2)	**0.001**
At 1 year	Total (n=1198)	Traj1 (n=342)	Traj2 (n=621)	Traj3 (n=235)	*P* value
Aortic valve area, cm^2	1.72 (1.43–2.04)	1.71 (1.44–2.03)	1.72 (1.43–2.05)	1.72 (1.46–2.01)	0.918
Peak aortic jet velocity, m/s	2.14 (1.84–2.50)	2.18 (1.87–2.50)	2.11 (1.81–2.48)	2.17 (1.85–2.54)	0.375
Mean transvalvular gradient, mmHg	9 (7-13)	9 (7-13)	9 (6-12)	10 (7-13)	0.297
LVEF, %	63.7 (58.7–68.1)	64.3 (59.0–68.4)	63.8 (58.8–68.2)	62.6 (58.4–67.3)	0.169
LVMI, g/m^2	107.4 (88.9–129.5)	105.0 (88.4–129.9)	107.6 (88.0–129.1)	113.1 (91.9–130.5)	0.185
Moderate or greater AR	6 (0.5)	2 (0.6)	4 (0.6)	0 (0.0)	0.652
Moderate or greater MR	68 (5.7)	23 (6.7)	35 (5.6)	10 (4.3)	0.451
Moderate or greater TR	86 (7.2)	33 (9.6)	40 (6.4)	13 (5.5)	0.100
At 2 years	Total (n=765)	Traj1 (n=224)	Traj2 (n=392)	Traj3 (n=149)	*P* value
Aortic valve area, cm^2	1.70 (1.44–2.06)	1.64 (1.39–1.96)	1.73 (1.45–2.06)	1.75 (1.49–2.12)	0.082
Peak aortic jet velocity, m/s	2.07 (1.78–2.44)	2.09 (1.77–2.44)	2.07 (1.78–2.42)	2.00 (1.77–2.45)	0.940
Mean transvalvular gradient, mmHg	12 (8-18)	12 (8-16)	12 (8-18)	13 (8-19)	0.494
LVEF, %	63.4 (59.0–67.8)	63.9 (60.1–68.2)	63.4 (58.8–67.5)	63.4 (58.6–67.8)	0.335
LVMI, g/m^2	104.2 (86.7–125.2)	104.4 (87.8–124.5)	100.9 (85.1–124.5)	112.2 (91.7–126.9)	0.034

Moderate or greater AR	6 (0.8)	3 (1.4)	2 (0.5)	1 (0.7)	0.489
Moderate or greater MR	37 (4.8)	13 (5.8)	18 (4.6)	6 (4.0)	0.696
Moderate or greater TR	73 (9.5)	22 (9.8)	44 (11.2)	7 (4.7)	0.070

At 3 years	Total (n=503)	Traj1 (n=145)	Traj2 (n=263)	Traj3 (n=95)	*P* value
Aortic valve area, cm^2	1.76 (1.43–2.09)	1.70 (1.40–2.08)	1.79 (1.48–2.09)	1.74 (1.42–2.08)	0.513
Peak aortic jet velocity, m/s	2.08 (1.78–2.41)	2.09 (1.84–2.41)	2.09 (1.75–2.41)	2.01 (1.78–2.38)	0.668
Mean transvalvular gradient, mmHg	9 (6–12)	9 (7–12)	9 (6–12)	8 (6–11)	0.516
LVEF, %	62.5 (58.3–67.0)	64.2 (60.0–68.2)	61.9 (58.0–66.1)	62.5 (58.2–67.2)	**0.023**
LVMI, g/m^2	104.6 (89.0–124.6)	103.9 (89.5–120.1)	103.8 (85.4–122.7)	111.0 (93.1–130.7)	0.123
Moderate or greater AR	3 (0.6)	3 (2.1)	0 (0.0)	0 (0.0)	0.030
Moderate or greater MR	25 (5.0)	9 (6.2)	12 (4.6)	4 (4.3)	0.715
Moderate or greater TR	40 (7.9)	17 (11.7)	18 (6.8)	5 (5.3)	0.121

At 4 years	Total (n=350)	Traj1 (n=97)	Traj2 (n=186)	Traj3 (n=67)	*P* value
Aortic valve area, cm^2	1.77 (1.46–2.13)	1.82 (1.50–2.07)	1.72 (1.44–2.17)	1.85 (1.48–2.11)	0.719
Peak aortic jet velocity, m/s	2.05 (1.78–2.41)	2.07 (1.79–2.49)	2.08 (1.79–2.40)	2.01 (1.70–2.25)	0.391
Mean transvalvular gradient, mmHg	9 (6–12)	8 (6–13)	9 (6–12)	8 (6–10)	0.255
LVEF, %	63.5 (56.0–67.3)	62.9 (58.6–66.4)	63.8 (59.2–67.6)	63.8 (60.1–67.3)	0.292
LVMI, g/m^2	109.6 (87.5–133.2)	111.5 (86.0–131.6)	106.2 (87.5–127.3)	115.4 (92.0–136.8)	0.293

Moderate or greater AR	5 (1.4)	2 (2.1)	3 (1.6)	0 (0.0)	0.713
Moderate or greater MR	26 (7.4)	8 (8.2)	14 (7.5)	4 (6.0)	0.859
Moderate or greater TR	37 (10.6)	21 (21.6)	12 (6.4)	4 (6.0)	**<0.001**
At 5 years	Total (n=202)	Traj1 (n=53)	Traj2 (n=109)	Traj3 (n=40)	*P* value
Aortic valve area, cm^2	1.81 (1.42–2.08)	1.83 (1.37–2.08)	1.81 (1.43–2.11)	1.80 (1.44–2.05)	0.875
Peak aortic jet velocity, m/s	2.00 (1.68–2.40)	2.07 (1.78–2.51)	2.02 (1.71–2.40)	1.81 (1.49–2.17)	**0.022**
Mean transvalvular gradient, mmHg	8 (5–12)	9 (6–13)	8 (6–12)	6 (4–10)	**0.030**
LVEF, %	63.1 (59.0–66.9)	63.2 (58.6–67.4)	62.4 (59.0–66.5)	63.0 (59.5–67.0)	0.731
LVMI, g/m^2	105.1 (87.9–126.9)	104.1 (90.3–126.4)	104.0 (89.3–123.5)	107.0 (85.1–133.9)	0.816
Moderate or greater AR	6 (3.0)	1 (1.9)	3 (2.8)	2 (5.0)	0.734
Moderate or greater MR	18 (9.0)	6 (11.3)	6 (5.6)	6 (15.0)	0.145
Moderate or greater TR	25 (12.4)	9 (17.0)	10 (9.3)	6 (15.0)	0.325

The number of patients analyzed at each follow-up time point reflects those with available echocardiographic data at that time point. Values are presented as median (interquartile range) or n (%), unless otherwise indicated. Traj1, Traj2, and Traj3 represent the low-level, intermediate-level, and high-level AIP trajectory groups, respectively. Abbreviations: AIP, atherogenic index of plasma; AR, aortic regurgitation; LVEF, left ventricular ejection fraction; LVMI, left ventricular mass index; MR, mitral regurgitation; PPM, prosthesis–patient mismatch; TR, tricuspid regurgitation.
